# Symptom duration is associated with failure of periprosthetic joint infection treated with debridement, antibiotics and implant retention

**DOI:** 10.3389/fsurg.2022.913431

**Published:** 2022-08-31

**Authors:** Hongyi Shao, Rui Li, Wang Deng, Baozhan Yu, Dejin Yang, Yixin Zhou, Jiying Chen

**Affiliations:** ^1^ Department of Orthopaedic Surgery, Beijing Jishuitan Hospital, Fourth Clinical College of Peking University, Beijing, China; ^2^ Senior Department of Orthopedics, the Fourth Medical Center of PLA General Hospital, Beijing, China; ^3^ Department of Orthopedics, the First Medical Center of PLA General Hospital, Beijing, China; ^4^ Department of Orthopaedics, No.2 Hospital of Baoding, Baoding, Hebei, China

**Keywords:** knee, hip, total joint arthroplasty, periprosthetic joint infection, debridement antibiotics implant retention, symptom duration

## Abstract

**Background:**

Debridement, antibiotics, and implant retention (DAIR) is an alternative treatment strategy for periprosthetic joint infection (PJI). However, no consensus exists regarding which patient population(s) may be most suitable for DAIR. This study aims to investigate the overall infection control rate and explore the prognostic factors associated with acute, hematogenous, and chronic PJIs treated with DAIR.

**Methods:**

We retrospectively reviewed the included patients who were diagnosed with PJI and underwent DAIR at two institutions from 2009 to 2018 (*n *= 104). We collected the clinical data, including demographics, preoperative laboratory tests, Charlson Comorbidity Index, surgical information, and culture organism results. Treatment success was defined according to the criteria reported by Diaz-Ledezma. All patients were followed for at least one year unless failure preceded that time point. A multivariable analysis was utilized to identify prognostic factors associated with treatment, and a Kaplan-Meier survival analysis was used to depict the infection control rate.

**Results:**

The overall treatment success rate in the current cohort of patients was 67.3% at a median 38.6 (interquartile range: 23.5, 90.7) months follow-up. Patients with a duration of infectious symptoms of more than ten days were more likely to fail (*P = *0.035, hazard ratio 8.492, 95% confidence interval 1.159–62.212). There was no difference among acute, hematogenous, and chronic infections in terms of failure rate (*P *= 0.161).

**Conclusions:**

DAIR is a reasonable treatment option for PJI, and its use in the setting of chronic infection does not appear to be a contraindication. Performing DAIR within ten days of the presentation of symptoms had a higher rate of treatment success.

## Introduction

Total joint arthroplasty (TJA) is a successful surgery for relieving pain and improving function in patients when extensive joint destruction occurs (1). The number of TJA procedures has increased in the past 20 years, and periprosthetic joint infection (PJI) is a devastating complication that can occur (2). PJI can result in higher morbidity and mortality and represents a substantial financial burden to both patients and society (3).

Most surgeons utilize a two-stage revision protocol to treat PJI (4). Despite this, some concerns exist, including high rates of morbidity and mortality (5, 6), a long interval time of disability (7), and increased cost (8). For these reasons, surgeons have pursued prosthesis retention as a possible treatment option for PJI. PJI can be divided into acute, hematogenous, and chronic infection based on the time from index surgery and duration of symptoms. Initially, the failure rate associated with debridement, antibiotics use (including systemic or topical use), and implant retention (DAIR) procedures was high, even when applied to settings of acute or acute hematogenous infections (9, 10). With improvement in surgical technique in conjunction with more effective antibiotic protocols, the success rates of DAIR procedures have been significantly improved in more recent reports (11, 12).

Patient selection is of paramount importance when considering a DAIR procedure. Previous studies have investigated prognostic factors for success, including soft tissue status, patient comorbidities, type of bacteria, and other factors (13–15). However, the success rate of DAIR in the treatment of PJI varied greatly, ranging from 31%–66%. Among these studies, most groups only performed a DAIR procedure in the setting of an acute or acute hematogenous infection; the cutoff time for surgical intervention remains controversial (16). Koyonos et al. (17) extended the indications for a DAIR procedure to chronic infection, but reported the failure rate up to 72%. Currently, there does not appear to be an absolute contraindication to performing DAIR in PJI patients with a stable prosthesis (18). Despite this, no guidance regarding patient selection heretofore exists for utilizing DAIR in acute, hematogenous, and chronic PJIs.

In order to evaluate the success rate of DAIR in the setting of acute, hematogenous, and chronic cases of PJI, and explore associated prognostic factors, the following questions were devised: (i) what was the infection control rate associated with the DAIR procedure in our cohort patients? (ii) was there any difference(s) among the varying types of infections? And (iii) what are the prognostic factors associated with treatment failure after DAIR?

## Methods

### Study population

After the institutional review boards of Beijing Jishuitan Hospital and Chinese PLA general hospital approved this study (S2020-056-01), we retrospectively reviewed the electronic medical records at two separate institutions from 2009 to 2018. The inclusion criteria were patients diagnosed with PJI according to the Musculoskeletal Infection Society (MSIS) criteria (19); patients who underwent the treatment of DAIR protocol; the follow-up time was at least one year unless the clinical failure was diagnosed prior to that time point. If a patient underwent more than one DAIR procedure, the information on the index procedure was included and then categorized into clinical failure after DAIR. Patients were excluded in cases where the index surgery included a revision for PJI or in primary cases of septic arthritis; or megaprosthesis, which replaces part of the femur or tibial, was used to reconstruct hip or knee. The surgeons decided on all the DAIR procedures according to the patients’ condition at that time, except that loosening or instability of the prosthesis was an absolute contraindication. In total, 112 patients were identified. After excluding six patients with isolated superficial infection who underwent superficial debridement without arthrotomy and two patients with mega-prostheses, 104 patients were eligible for the current study.

### Treatment protocol

There was an infectious disease team in each hospital. Patients were selected for DAIR based on an individualized discussion dependent on each patient's unique situation, including symptoms, soft tissue status, medical comorbidities, and whether prothesis was stable or not. Several fellowship-trained surgeons familiar with the DAIR procedure performed and implemented the protocols at these two institutions, and all patients were treated according to the same therapeutic protocol. A posterolateral approach was utilized for hips, and a midline incision with a medial parapatellar arthrotomy was utilized for knees. If a sinus tract was present, it would be excised intra-operatively. During the procedure, 3–5 samples, including synovial fluid and tissue, were sent for culture and synovial fluid analysis to both confirm an infection and guide antibiotic use after surgery. During the debridement, hydrogen peroxide, saline, iodine, and saline were successively used for joint lavage. The amount of saline utilized was at least 10 L in each case. The treating surgeon decided to retain or exchange the modular components (polyethylene for knees or liner and femoral head for hips) intra-operatively. In most cases, the modular part would be replaced for stability and thorough debridement. However, the modular part would be retained if it is difficult to take out and the joint was stable. We then re-draped the surgical site before inserting the new modular component and suturing the wound.

Given that DAIR is considered an urgent surgery, even we aspirated every joint before DAIR procedure, only 19 of the 104 cases had culture results pre-operatively. Therefore, sensitive antibiotics were used for positive culture cases before the DAIR procedure, otherwise, vancomycin and a third-generation cephalosporin were combined to cover both gram-positive and negative bacteria initially. We routinely used these broad-spectrum antibiotics prior to culture results returning from the lab. Once the results were received, the antibiotic regimen was narrowed in an organism-specific fashion, except in cases where cultures remained negative (20). Patients received intravenous antibiotics for at least two weeks, then converted to an oral regimen for at least an additional four weeks. Topical antibiotics were given routinely. Sensitive antibiotics were given if the culture was positive before the DAIR procedure, otherwise vancomycin was given topically. Patients were administered antibiotics systemically for no more than three months after the DAIR procedure. If the patient used antibiotics continuously for more than three months, we would check the follow-up medical record. We categorize it as a treatment failure if the patient still has infectious symptoms.

### Data collection and outcome assessment

The medical records of all patients were reviewed for information, including gender, age, height, and weight at the time of the DAIR procedure. Comorbidities were assessed using the Charlson Comorbidity Index (CCI), in addition to whether the patients had diabetes mellitus or rheumatoid arthritis. We also collected preoperative laboratory results, including serum C reactive protein (CRP), hemoglobin, albumin, and perioperative culture results. According to current clinical practice and previous studies (21–23), the continuous variables were categorized into groups (age ≥60 years and <60 years; BMI ≥35 kg/m^2^ and <35 kg/m^2^; CCI ≥4 and <4; CRP ≥115 mg/L and <115 mg/L; hemoglobin ≥110 g/L and <110 g/L and albumin ≥35 g/L and <35 g/L). We also recorded the type of index surgery (primary or revision and hip or knee), whether the patient had a sinus tract, and whether the modular components were retained or exchanged. The duration of clinical symptoms (e.g., fever, swelling, tenderness, wound drainage, etc.) was defined as the number of days from onset until the day that the DAIR procedure was performed. If patients had persistent symptoms after the index surgery, the duration of symptoms was from the date of the index surgery to the DAIR procedure. Acute infection was defined as a time period of <90 days from the index surgery to the DAIR procedure. If the time was >90 days while symptom duration was <3 weeks, this was defined as a hematogenous infection. If the time from the index surgery to the DAIR procedure was >90 days and the duration of symptoms was >3 weeks, then the infection was considered chronic (24). No statistical difference was detected in demographic data between the two institutions (Table 1).

**Table 1:** Demographic data between the two institutions.

Variable	Total	Institution 1	Institution 2	*P*-value
	*n* = 104	*n* = 43	*n* = 61	
Age^a^, year	62.5 (54, 75)	60 (54, 72)	64 (53, 74)	0.959
Male	45 (43.3%)	18 (41.9%)	27 (44.3%)	0.808
Weight^b^, kg	72.2 ± 14.1	70.5 ± 12.7	73.4 ± 15.0	0.306
BMI^a^, kg/m^2^	26.5 (23.2, 29.4)	25.9 (22.4, 29.8)	27.1 (23.4, 29.8)	0.302
Index surgery				
Primary knee	66 (63.5%)	25 (58.1%)	41 (67.2%)	0.053^c^
Primary hip	19 (18.3%)	8 (18.6%)	11 (18%)	
Revision knee	9 (8.7%)	2 (4.7%)	7 (11.5%)	
Revision hip	10 (9.63%)	8 (18.6%)	2 (3.3%)	
Infection type				
Acute	55 (52.9%)	26 (60.5%)	29 (47.5%)	0.398
Hematogenous	24 (23.1%)	9 (20.9%)	15 (24.6%)	
Chronic	25 (24.0%)	8 (18.6%)	17 (27.9%)	
Success case	70 (67.3%)	28 (65.1%)	42 (68.9%)	0.689

^a^ Data with a non-normal distribution are represented with the median (interquartile range).

^b^ Data with a normal distribution are represented with mean ± standard deviation; Continuous variables in demographic data (age, weight and BMI) were examined with use of independent t test (if data followed normal distribution) or Mann-Whitney U test (if data did not follow normal distribution) between two institutions. Categorical variables in demographic data (gender, infection type and success) were analyzed with use of either the Pearson chi-square test or the Fisher exact test between two institutions.

^c^ Fisher’s exact test.

Following completion of treatment, patients were encouraged to return for routine follow-up appointments at three months, six months, and 1-year post-operatively, and then annually after that. Treatment success was defined as the eradication of infection without persistent clinical signs or symptoms. We used the treatment failure criteria, as defined by Diaz-Ledezma (25), which incorporated (1) a fistula, drainage, or pain, and infection recurrence caused by the same organism; (2) subsequent surgical intervention for infection after surgery; and/or (3) occurrence of PJI-related mortality.

### Statistical analysis

Univariate analyses were performed to identify potential risk factors for the failure of DAIR. Continuous variables with normal distribution were presented as mean and standard deviation (SD) and were compared between groups using the Student's t-test. Non-normally distributed continuous variables were presented as medians and quartiles and were compared between groups using the Mann–Whitney *U* test. Categorical variables were compared between groups using the chi-square test or Fisher exact test. Variables with a *P*-value <0.1 in univariate analyses were then included in the subsequent multivariable analysis.

**Figure A1:**
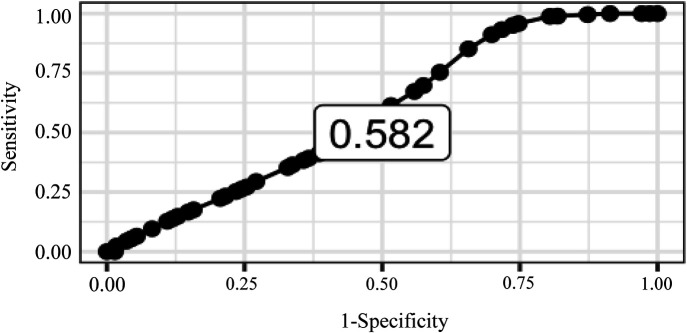
Time-dependent ROC to identify the cutoff value for duration of infection symptoms (days). We used 5 years as the predict point of time, the ROC curve got the maximal AUC (0.582) and the cutoff value is reported as 10 days.

A multivariable analysis was performed by using the Cox proportional hazards regression model. Given that the most optimal cutoff value for the duration of infectious symptoms was uncertain, a time-dependent ROC (Receiver Operating Characteristic) was applied to assess this with a Kaplan-Meier method (Appendix Figure A1). Ultimately, ten days was determined to be the cutoff value at which a difference could be detected, and so this was utilized accordingly.

In addition, Kaplan-Meier survival analysis was utilized to depict overall infection control in this cohort of patients, and Breslow tests were used to compare the success rate of DAIR among acute, hematogenous, and chronic PJI cases.

Significance was set at *P*-value <0.05. All statistical analyses were conducted with IBM SPSS (version 22.0 for Windows; SPSS Inc., Chicago, IL, USA) except for the time-dependent ROC, which was conducted with R software (version 3.6.2; Survival ROC package; R Foundation for Statistical Computing, Vienna, Austria). In addition, a power analysis was conducted by PASS software (version 15.0), primarily based on the analysis of symptom duration, under the assumption of a two-sided type 1 error rate of 5%, and has 80% power to show a clinically significant advantage, the required sample sizes were 40, which is less than our actual sample size. Power analysis for multivariate cox regression showed enough power (0.995).

## Results

**Figure 1:**
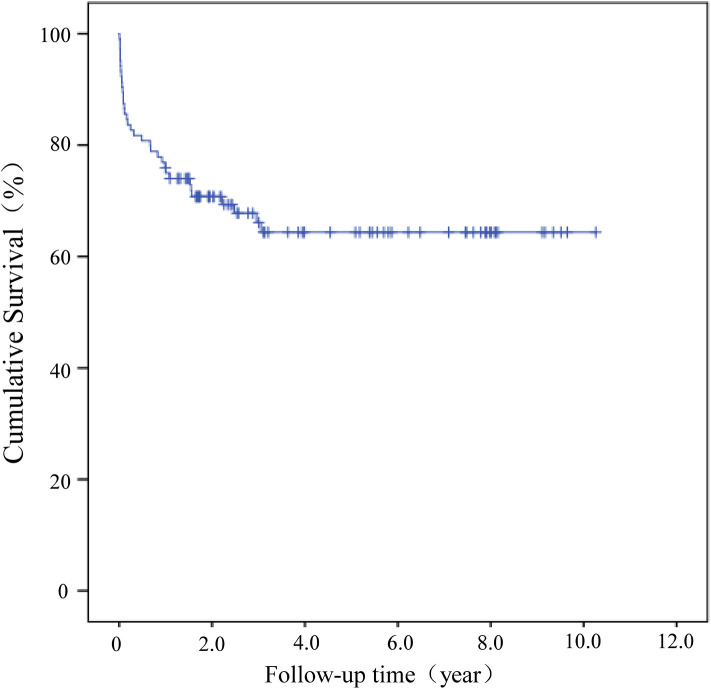
Kaplan–Meier (KM) survival analysis for the cohort of patients who underwent a DAIR procedure failure was defined as the endpoint.

The median follow-up time for patients in this cohort was 38.6 (interquartile range: 23.5, 90.7) months. Among them, 67 patients achieved treatment success at the final follow-up. Three patients died beyond their respective one-year follow-up time points for reasons that were objectively unrelated to infection, and we, therefore, categorized them into the group of treatment success. Thus, 34 patients met the criteria for treatment failure, and the overall success rate was 67.3% at the time of final follow-up. Time to treatment failure ranged from 3 days to 37.2 months post-operatively. The cumulative success rate was 76.9% (95% confidence interval (CI), 69.2%–85.5%) at one year and 64.4% (95% CI, 57.1%–76.6%) at five years follow-up (Figure 1). Among the cases of treatment failure, eight patients underwent no further surgery and were prescribed antibiotic suppression due to medical comorbidity(ies) or a reluctance to accept further surgery. Seven patients received repeat DAIR, and 3 of them failed. A total of 19 patients underwent a one or two-stage revision procedure, of which 13 succeeded.

After a regression analysis model was established, it was identified that a longer duration of symptoms was related to the failure of a DAIR procedure. Univariate analysis revealed that the *P*-values of CRP, modular component exchange, duration of symptoms, and different types of infection were <0.1 for treatment failure and were entered into the Cox proportional hazards regression model (Table 2). Multivariate analysis revealed that only a longer duration of symptoms was identified as an independent predictor of treatment failure. The hazard of failure for a patient with infectious symptoms for more than ten days was nearly 8.5 times the hazard for patients with less than ten days of symptoms. No statistical significance was detected with the other factors (Table 3).

**Table 2:** Comparison of demographics, medical, surgical information and culture results with a univariate analysis.

		Success Rate (*n* = 70)	Failure Rate (*n* = 34)	*P*-value
Demographics				
Age	≥60 years	45 (72.6%)	17 (27.4%)	
	<60 years	25 (59.5%)	17 (40.5%)	0.164
Gender	Male	27 (60.0%)	18 (40.0%)	
	Female	43 (72.9%)	16 (27.1%)	0.165
BMI	≥35 kg/m^2^	1 (33.3%)	2 (66.7%)	
	<35 kg/m^2^	69 (68.3%)	32 (31.7%)	0.249^a^
DM	Yes	12 (75.0%)	4 (25.0%)	
	No	58 (65.9%)	30 (34.1%)	0.476
Rheumatoid arthritis	Yes	4 (66.7%)	2 (33.3%)	
	No	66 (67.3%)	32 (32.7%)	1.000^a^
CCI	≥4	31 (72.1%)	12 (27.9%)	
	<4	39 (63.9%)	22 (36.1%)	0.382
Preoperative tests				
CRP	≥115 mg/L	22 (81.5%)	5 (18.5%)	*** ***
	<115 mg/L	48 (62.3%)	29 (37.7%)	***0***.***068***
Hemoglobin	≥110 g/L	24 (70.6%)	10 (29.4%)	
	<110 g/L	46 (65.7%)	24 (34.3%)	0.619
Albumin	<35 g/L	23 (67.6%)	11 (32.4%)	
	≥35 g/L	47 (67.1%)	23 (32.9%)	0.302
Surgical information				
Sinus	Yes	32 (62.7%)	19 (37.3%)	
	No	38 (71.7%)	15 (28.3%)	0.331
Modular part exchange	Yes	54 (73.0%)	20 (27.0%)	*** ***
	No	16 (53.3%)	14 (46.7%)	***0***.***053***
Duration of symptoms	≥10 days	50 (60.2%)	33 (39.8%)	*** ***
	<10 days	20 (95.2%)	1 (4.8%)	***0***.***002***
Types of infection	Acute	40 (72.7%)	15 (27.3%)	
	Hematogenous	18 (75.0%)	6 (25.0%)	
	Chronic	12 (48.0%)	13 (52.0%)	***0***.***060***
Types of index surgery	Primary knee	48 (72.7%)	18 (27.3%)	
	Primary hip	11 (57.9%)	8 (42.1%)	
	Revision knee	6 (66.7%)	3 (33.3%)	
	Revision hip	5 (50.0%)	5 (50.0%)	0.118
Organism	Staphylococcus (MR)	13 (59.1%)	9 (40.9%)	
	Staphylococcus (MS)	5 (62.5%)	3 (37.5%)	
	Gram-negative	7 (63.6%)	4 (36.4%)	
	Polymicrobial	8 (72.7%)	3 (27.3%)	
	Culture negative	24 (68.6%)	11 (31.4%)	
	others	13 (76.5%)	4 (23.5%)	0.903

BMI, body mass index; DM, diabetes mellitus CCI, charlson comorbidity index; CRP, C-reactive protein; MR, methicillin-resistant; MS, methicillin-sensitive.

^a^ Fisher’s exact test.

The bold values mean it has statistical difference.

**Table 3:** Multivariable analysis for treatment failure following DAIR for PJI cases.

Variables	Category	Hazard Ratio	95% CI	*P*-value
CRP	<115 mg/L	reference		
	≥115 mg/L	1.374	0.510–3.697	0.530
Modular part exchange	Yes	reference		
	No	1.533	0.771–3.051	0.223
Duration of symptoms	<10 days	reference		
	≥10 days	8.492	1.159–62.212	***0***.***035***
Types of infection	Acute	reference		
	Hematogenous	1.610	0.605–4.290	0.340
	Chronic	1.755	0.832–3.700	0.140

CRP, C-reactive protein.

The bold values mean it has statistical difference.

**Figure 2:**
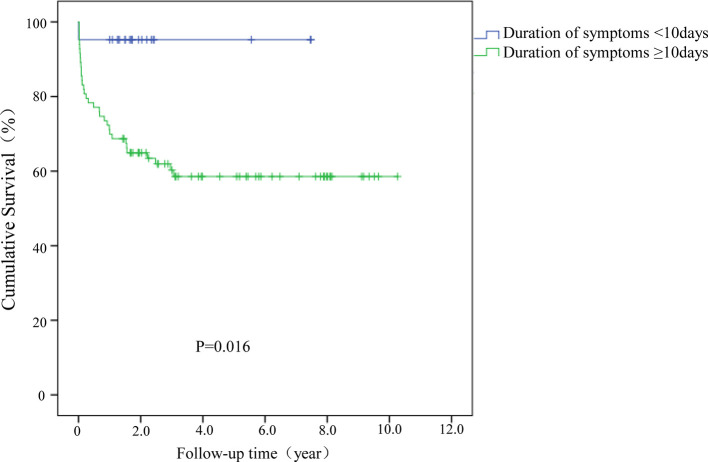
Kaplan–Meier (KM) survival analysis for cases with symptom duration greater or less than 10 days.

**Figure 3:**
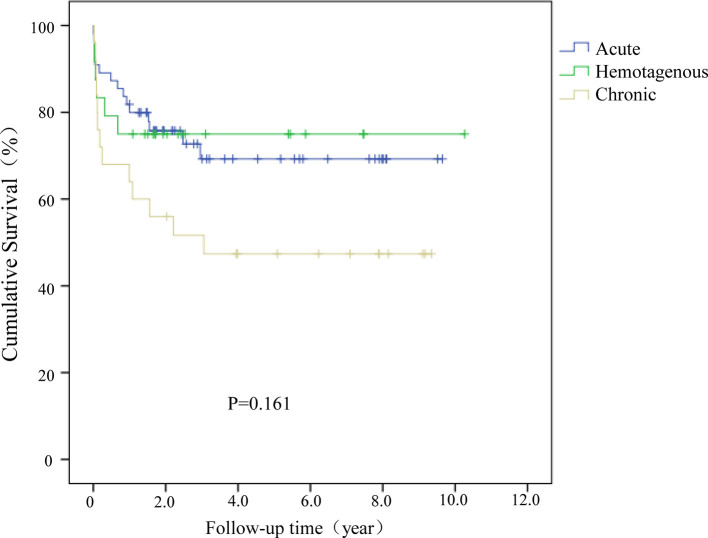
Kaplan–Meier (KM) survival analysis for acute, hematogenous, and chronic cases of PJI.

A Kaplan-Meier (KM) survival analysis was further performed to compare results between treatment groups. Patients were sub-grouped by symptom duration, and failure of a DAIR procedure was defined as the endpoint, and results demonstrated that the survivorship of these cases performed more than ten days after symptoms were lower than those performed within ten days (*P *= 0.016; Figure 2). No statistical difference was detected among acute, hematogenous, and chronic cases of PJI (*P *= 0.161; Figure 3).

## Discussion

The current study included two centers and more than 100 cases of acute, hematogenous, and chronic infections treated as DAIR procedure with an overall infection control (e.g., treatment success) rate of 67.3% at final follow-up. There was no difference in terms of predicting infection control among different types of infection, and symptom duration of fewer than ten days was more predictive of success.

Infection control is an important priority when surgeons choose to perform a DAIR procedure as a means of managing PJI. Kunutsor et al. performed a systematic review that included 4,897 cases treated with DAIR and reported an overall infection control rate of 61.4%, with a mean follow-up time of 3.6 years (11). This was similar to the infection control rate reported in the current study. However, a subgroup analysis looking at cases performed before or after the year 2000 revealed a higher infection control rate of 65.0% in the current century as opposed to the prior with an infection control rate of 51.5%. The potential reasons for this observation may be improvements in surgical technique, efficiency in bacterial culture, and optimization in the use of antibiotics. Although most surgeons utilize a DAIR protocol for acute or hematogenous infections, a previous consensus recommendation did not advise against using a DAIR procedure except in cases with evidence of prosthetic loosening (18). As a result, expanding the boundary of indications for DAIR was pursued.

In 2011, Koyonos et al. (17) compared acute, hematogenous, and chronic PJIs treated with DAIR, although no differences were detected among the groups, the infection control rates among all three groups were lower than 50%. Grammatopoulos et al. (26) separately reported a cohort of PJI cases managed with DAIR, including both acute and chronic infections. Their overall infection control rate was 84% and higher than the percentage reported in the current study. In their study, when the cutoff time from the index surgery to the DAIR procedure was between 4 and 13 weeks, the infection control rate revealed no statistical difference. The infection control rates of acute, hematogenous, and chronic PJIs in the cohorts of the current study were 72.8% (40/55), 75.0% (18/24), 48.0% (12/25), respectively, which were without statistical difference. Notably, we also performed a secondary analysis in which we differentiated acute from chronic infections with a cutoff of 4 weeks, and there remained no statistically significant difference among the groups (Appendix Table A1).

Identifying the optimal cutoff time for differentiating acute from chronic is difficult, but may help define a paradigm of communication amongst practitioners in the field (16). Fehring et al. reported that the infection control rate of DAIR had no statistical difference when the cutoff was 30 or 90 days (27), to which our results were similar. However, although there was no statistical difference for infection control rate between chronic and acute infection according to different standards. The infection control rate of chronic infection was still lower than that of acute and hematogenous infection. Chronic infection means a longer time of infection which may be related to biofilm formation. That would compromise the results of DAIR (16). While acute and hematogenous infection has less bacteria of biofilm form, which is easier to be eradicated. Why we did not find the statistical difference among acute, hematogenous, and chronic infections may be related to the limited number of cases included in this study. At the same time, it may also be that more suitable patients were selected during the selection of DAIR. And the cutoff time is not directly related to bacteria, or host factors. Different patient selection, surgical techniques and/or other confounders may also explain why our study had different infection control rates from others currently available in the literature.

In this study, having a duration of infectious symptoms longer than ten days was the only independent risk factor that was detected for failing to establish infection control with DAIR. Several previous studies have reported that a shorter duration of symptoms was related to the eradication of infection (28–31). Surgical debridement and subsequent continuous antibiotics may remove planktonic bacteria and younger biofilm (32). A longer duration of symptoms theoretically indicates higher rates of biofilm formation and may explain why a longer duration of symptoms infers higher failure from DAIR. However, among these studies, the optimal duration of symptoms in terms of days for DAIR was variable. Fink et al. (31) reported that the target symptom duration was two days, while Narayanan et al. (30) reported that it was two weeks. Limited cases or a lack of reliable statistical methods may explain this difference. Other studies (9, 15, 22) were unable to detect a longer duration of symptoms as a predictor of failure for DAIR. However, all of these studies included only acute or acute hematogenous PJIs, which indicates that the duration of symptoms in all patients was inherently short. The multicenter study from Lowik et al. (15) contained a large cohort of 386 patients, and all had symptom duration of <21 days, thereby preventing any true analysis for symptoms beyond that time point. In contrast, our study combined acute, hematogenous, and chronic infections, each with objectively different durations of symptoms. The statistical method of a time-dependent ROC to identify an optimal cutoff time made our results particularly robust.

Other known risk factors, such as the presence of a sinus tract (15), modular component exchange (26), or staphylococcal infections (13, 17), are reportedly related to the failure of DAIR. Despite that, in the current cohort, although the infection control rates of patients identified with these particular factors were lower than the overall population, we failed to detect statistical significance. The key to the success of DAIR lies in biofilm removed through mechanical and chemical disruption (33). The minimum biofilm eradication concentration (MBEC) is much higher than the minimum inhibitory concentration (MIC) for planktonic bacteria (34). We added local antibiotics intraoperative, which may provide better clinical outcomes (35, 36). Besides that, individual antibiotic selection under the guidance of multidisciplinary specialists, an improvement in surgical techniques, and strict patient selection can all represent potential reasons as to why the current study had reasonable infection control and was unable to detect differences in these factors.

There are several limitations to be acknowledged in this study. First, it was a retrospective study which indicates inherent weaknesses. However, DAIR is an urgent surgery and, therefore, challenging to organize a prospective study. Both institutions in the current study had an assigned person to crosscheck the data to ensure reliability. Even so, due to the great subjectivity in the selection of DAIR, there was still be some heterogeneity in the data. Secondly, the duration of symptoms was subjective and dependent on the patient’s description. Nevertheless, patients were sensitive to symptoms, including fever, wound drainage, swelling, and tenderness, and surgeons were invested in taking a careful patient history. Third, the case number is limited, especially in some demographic factors, including those with a BMI higher than 35 kg/m^2^; this may yield type I error. With the exception of registry data, the patient numbers in a study assessing DAIR are unlikely to be significant. The current study reported the largest cohort of data in our region, and further collaborative studies should be pursued. Finally, although some cases had relatively short follow-up time, most cases of failure occurred relatively close to the DAIR procedure itself and are thus still likely to capture our clinical endpoint.

## Conclusion

In conclusion, DAIR is a reasonable treatment option for PJI, and chronic infection does not appear to be a contraindication, with a 48% success rate in this cohort of patients. Performing DAIR within a period in which the duration of symptoms was less than ten days achieved a satisfactory clinical result in most cases. Further investigation with a larger number of cases and longer follow-up time points may strengthen these clinical findings.

## Data Availability

The raw data supporting the conclusions of this article will be made available after request and approved by IRB of both hospitals.

## Appendix

APPENDIX FIGURE A1

APPENDIX TABLE A1

**Table A1:** Compare the failure rate of different types of infection with the cut off time as 4 weeks.

	Success (*n *=* *70)	Failure (*n *=* *34)	*p*-value
Acute	16 (22.9%)	9 (26.5%)	
Hematogenous	31 (44.3%)	9 (26.5%)	
Chronic	23 (32.9%)	16 (47.1%)	0.198

Acute infection: the time between DAIR and index surgery was less than 4 weeks; Hematogenous infection: the time between DAIR and index surgery was more than 4 weeks while the duration of infectious symptoms was less than 3 weeks; Chronic infection: the time between DAIR and the index surgery was more than 4 weeks while the duration of infectious symptoms was more than 3 weeks.
